# Extracellular Adenosine Triphosphate (eATP) and Its Metabolite, Extracellular Adenosine (eAdo), as Opposing “Yin–Yang” Regulators of Nlrp3 Inflammasome in the Trafficking of Hematopoietic Stem/Progenitor Cells

**DOI:** 10.3389/fimmu.2020.603942

**Published:** 2021-01-29

**Authors:** Mariusz Z. Ratajczak, Magda Kucia

**Affiliations:** ^1^Stem Cell Institute at Division of Hematology, Department of Medicine and James Graham Brown Cancer Center, University of Louisville, KY, United States; ^2^Center for Preclinical Studies and Technology, Department of Regenerative Medicine Medical University of Warsaw, Warsaw, Poland

**Keywords:** NOD-like receptor family pyrin domain-containing protein 3 (Nlrp3) inflammasome, stem cell mobilization, stem cell homing and engraftment, extracellular ATP, purinergic signaling

## Abstract

Nlrp3 inflammasome plays a pleiotropic role in hematopoietic cells. On the one hand, physiological activation of this intracellular protein complex is crucial to maintaining normal hematopoiesis and the trafficking of hematopoietic stem progenitor cells (HSPCs). On the other hand, its hyperactivation may lead to cell death by pyroptosis, and prolonged activity is associated with sterile inflammation of the BM and, as a consequence, with the HSPCs aging and origination of myelodysplasia and leukemia. Thus, we need to understand better this protein complex’s actions to define the boundaries of its safety window and study the transition from being beneficial to being detrimental. As demonstrated, the Nlrp3 inflammasome is expressed and active both in HSPCs and in the non-hematopoietic cells that are constituents of the bone marrow (BM) microenvironment. Importantly, the Nlrp3 inflammasome responds to mediators of purinergic signaling, and while extracellular adenosine triphosphate (eATP) activates this protein complex, its metabolite extracellular adenosine (eAdo) has the opposite effect. In this review, we will discuss and focus on the physiological consequences of the balance between eATP and eAdo in regulating the trafficking of HSPCs in an Nlrp3 inflammasome-dependent manner, as seen during pharmacological mobilization from BM into peripheral blood (PB) and in the reverse mechanism of homing from PB to BM and engraftment. We propose that both mediators of purinergic signaling and the Nlrp3 inflammasome itself may become important therapeutic targets in optimizing the trafficking of HSPCs in clinical settings.

## Introduction

NLR family pyrin domain-containing protein 3 (NLRP3) is a component of the innate immune system that functions as a pattern-recognition receptor (PRR) that recognizes both endogenous danger-associated molecular patterns (DAMPs), such as extracellular ATP (eATP), and certain pathogen-associated molecular patterns (PAMPs) (1–4). The responsiveness of Nlrp3 inflammasome to eATP makes this PRR unique among other cytoplasmic receptors from this family, including other members of the NOD-like receptor family, such as NOD1 and NOD2, as well as RIG-I-like receptors (5, 6).

The Nlrp3 inflammasome was initially found to be expressed in macrophages involved in triggering the immune response (7–9). However, recent results indicate that the Nlrp3 inflammasome is also expressed in other cell types, including hematopoietic stem/progenitor cells (HSPCs) (10–14). This should not be surprising, as macrophages originate later in the differentiation pathway from these stem cells. For many years, Nlrp3 inflammasomes’ role was associated with several pathologies, such as dominantly inherited auto-inflammatory diseases known as a cryopyrin-associated periodic syndrome or familial Mediterranean fever (15, 16). Moreover, the Nlrp3 inflammasome has been related to the pathogenesis of type 2 diabetes, multiple sclerosis, gout, hemorrhagic stroke, neuroinflammation, Alzheimer’s and Parkinson’s diseases, as well as atherosclerosis and even cancerogenesis (17–23). Also, as described in a recent elegant report, Nlrp3 inflammasome is activated in aged HSPCs due to mitochondrial stress contributing to the aging of these cells (10). The same group also reported that an acetylation switch of the Nlrp3 inflammasome regulates aging-associated chronic inflammation and insulin resistance (24). Finally, it is essential to pinpoint that hyperactivation of Nlrp3 inflammasome in hematopoietic cells may lead to cell damage and death in the mechanism of pyroptosis (25–27).

Recently, however, there have been additional biological functions proposed for the Nlrp3 inflammasome that are the main topic of this review, including involvement in regulating the normal trafficking of HSPCs during pharmacological mobilization, in which these cells egress from bone marrow (BM) into peripheral blood (PB), and in homing and engraftment to BM after transplantation (13, 14, 28). These hematopoietic effects were observed both directly in HSPCs and in non-hematopoietic cells in the BM microenvironment. Moreover, Nlrp3 inflammasome has been implicated in maintaining the pool of HSPCs in BM (29). This, however, requires further more detailed studies.

Based on these effects, accumulating evidence indicates that there are two sides to activation of Nlrp3 inflammasome in hematopoietic cells, a “good” side and a “bad” side. On the one hand, Nlrp3 inflammasome is vital in regulating physiological processes, such as the trafficking of HSPCs (30, 31), and on the other hand, if hyperactivated can contribute to adverse consequences leading to cell pyroptosis (25, 32). The Nlrp3 inflammasome is also involved in certain other hematological pathologies, including *i)* myelodysplastic syndrome, *ii)* myeloproliferative neoplasms, *iii)* leukemia, iv) graft-versus-host disease (GvHD) after transplantation, and *v)* cytokine storms as a complication of CAR-T cell therapy or COVID19 infection (24, 28, 33–39). The pleiotropic role of Nlrp3 inflammasome in hematopoiesis has been a subject of several excellent reviews (40–46). In this review, however, we will focus on the “good side” of the Nlrp3 inflammasome as a new positive regulator of stem cell trafficking and homeostasis of the BM microenvironment.

Based on those mentioned above, we need to better understand the actions of this intracellular protein complex to define the boundaries of the safety window and shed more light on the transition of the Nlrp3 inflammasome from being beneficial to detrimental.

## Mobilization of Hematopoietic Stem/Progenitor Cells from Bone Marrow Into Peripheral Blood and Their Homing and Engraftment After Transplantation to the Bone Marrow Microenvironment

HSPCs are tireless travelers as they migrate during embryogenesis, changing their micro-environmental locations to where hematopoiesis is active at a given age of development. This developmental migration begins from the yolk sac and aorta–gonad–mesonephros region, continues through a period of active hematopoiesis in the fetal liver, and finally reaches the developing BM (47–51). Nevertheless, throughout adult life, some percentage of HSPCs is detectable circulating in PB, on the way to finding new niches in other areas of BM or being “homeless” and awaiting elimination from the circulation.

HSPCs are retained in BM niches by the interaction of the α-chemokine stromal-derived factor 1 (SDF-1) and vascular adhesion molecule 1 (VCAM-1), expressed by cells in BM hematopoietic niches, with the CXCR4 receptor and the very late antigen 4 (VLA-4) integrin receptor, respectively, expressed on the surface of HSPCs (52–54). If HSPCs undergo symmetric division in stem cell niches, two new HSPCs are created, and as expected, one of them should leave the niche in order to find an available and supportive microenvironment. This could be a potential mechanism that maintains a constant number of HSPCs in areas of the BM where active hematopoiesis occurs. Despite the identification of some intracellular determinants that characterize cells undergoing symmetric versus asymmetric division, further studies are needed to elucidate at molecular level decisions made by dividing HSPCs and a role of the hematopoietic niche in these processes (55).

The number of these cells circulating in PB increases significantly during infections, tissue/organ damage, stress situations, and even after strenuous exercise (56). The forced egress of HSPCs from BM into PB is called mobilization, and a particular example is a pharmacological mobilization, which occurs after administration of pro-mobilizing drugs, such as the cytokine granulocyte-colony stimulating factor (G-CSF) or the small-molecule antagonist of the CXCR4 receptor (AMD3100). Pharmacological mobilization is a procedure employed in the clinic to enrich the PB for HSPCs, and these are subsequently harvested by leukapheresis for therapeutic applications (54, 57–60). The reverse mechanism, in which HSPCs, after being infused into PB during hematopoietic transplantation, migrate from PB to BM to reach stem cell niches, is called homing (61). This process is followed by engraftment and expansion of the transplanted cells.

Several years of intensive research have been dedicated to elucidating these phenomena at the cellular and molecular levels, and several complementary and redundant pathways have been proposed (62, 63). In this review, however, we will follow the accumulating evidence that both pharmacological mobilization and conditioning for hematopoietic transplantation by myeloablative chemo- or radiotherapy induce a state of sterile inflammation in the BM microenvironment (64). The former treatment stimulates the egress of HSPCs from BM into PB, while the latter treatment orchestrates HSPC homing to, and engraftment in, BM niches. An important role is played here by activation of innate immunity, including its cellular arm (involving monocytes, macrophages, granulocytes, and dendritic cells) and its humoral arm (involving the complement cascade [ComC] and soluble, circulating pattern-recognition receptors [PRRs]). Intracellularly, the Nlrp3 inflammasome is a crucial element of the innate immune system, which operates in cells belonging to the cellular arm of innate immunity (3) and, as recently demonstrated, is also expressed in HSPCs (12, 13, 30).

The Nlrp3 inflammasome is an important sensor of pathological changes that occur in the body and, as a PRR, responds to both endogenous danger-associated molecular pattern molecules (DAMPs) and pathogen-associated molecular pattern molecules (PAMPs) (1, 6, 65–67). It is important to emphasize that the Nlrp3 inflammasome has, among all intracellular PRRs or NOD-like receptors (NLRs), the unique property of being activated by extracellular adenosine triphosphate (eATP) that by engaging P2X7 and P2X4 purinergic receptors, induces K^+^ efflux and mitochondrial damage with mitochondrial reactive oxygen species (mtROS) release that activates Nlrp3 inflammasome (68–70). This makes the Nlrp3 inflammasome a crucial link between purinergic signaling and innate immunity responses (71, 72).

In addition to eATP, which is the central mediator of purinergic signaling and operates through P2X purinergic receptors, the Nlrp3 inflammasome can also be activated after stimulation by ComC cleavage fragments (the anaphylatoxins C3a and C5a and the non-lytic C5b-C9 membrane attack complex [MAC]), stromal-derived factor 1 (SDF-1), sphingosine-1-phosphate (S1P), or prostaglandin E2 (PGE2) (73–77). Interestingly, all these listed factors play an important role in the trafficking of HSPCs, which again supports the tight connection between Nlrp3 inflammasome activity and HSPC mobilization and homing. Another important indication is the fact that endotoxins, including liposaccharide (LPS), which on the one hand, potent inducers of HSPCs mobilization, on the other activate Nlrp3 inflammasome. As demonstrated in an elegant paper from Dr. Fibbe’s group, mice depleted of Gram-negative intestinal bacteria by metronidazole treatment or exposed to ploymyxin B in drinking water that binds and eliminates free LPS are poor mobilizers of HSPCs (78). Therefore, since these bacteria are a source of circulating LPS, which is crucial for the expression and basic priming of Nlrp3 inflammasome in cells, it further supports the role of this protein complex in HSPC mobilization (13). As reported, LPS may activate Nlrp3 inflammasome directly after binding to Toll-like receptor 4 (TLR4) *via* alternative pathway (79, 80).

### The Involvement of the NOD-Like Receptor Family Pyrin Domain-Containing Protein 3 Inflammasome in the Pharmacological Mobilization of Hematopoietic Stem/Progenitor Cells

**Figure 1:**
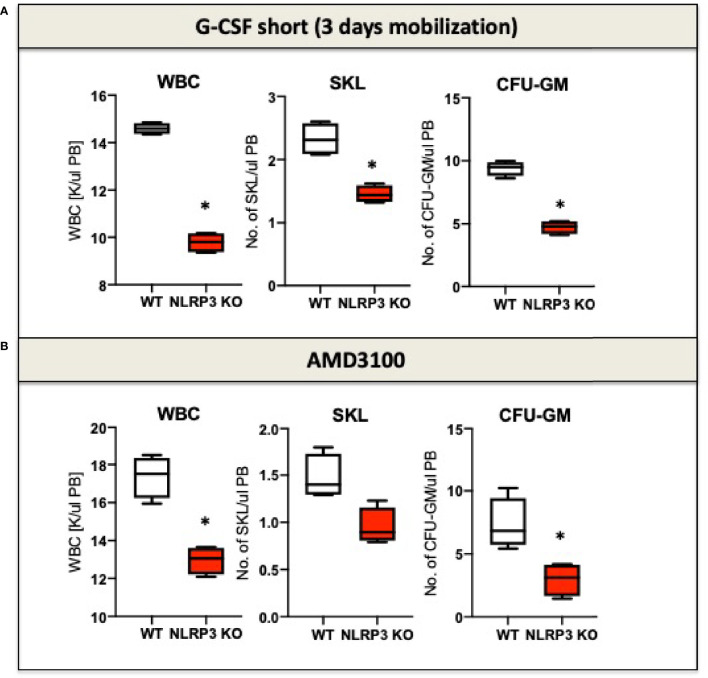
The impact of Nrlp3 KO on the mobilization of murine HSPCs. Mononuclear cells (MNCs) were isolated from WT and Nrlp3^–/–^ mice after 3 days of G-CSF **(A)** or AMD3100 **(B)** mobilization. The numbers of WBCs, SKL (Sca-1^+^/c-kit^+^/Lin^−^) cells, and CFU-GM clonogenic progenitors were evaluated in PB. Results from two independent experiments are pooled together. *p ≤ 0.01.

It is known that eATP is a chemoattractant for HSPCs (72). Moreover, as demonstrated recently, the Nlrp3 inflammasome is an important intracellular sensor that activates migration of HSPCs in response to significant chemoattractant for these cells, including stroma derived factor-1 (SDF-1), sphingosine-1 phosphate (S1P), and eATP (11, 12). To illustrate this, mice that are deficient in Nlrp3 (Nlrp3-KO mice) are poor mobilizers of HSPCs in response to commonly employed pro-mobilizing agents, such as G-CSF and AMD3100 (**Figure 1**). A similar effect is observed in normal mice exposed to a specific Nlrp3 inflammasome inhibitor, the small molecule MCC950 (12). At the cellular level, HSPCs from Nlrp3-KO mice also show attenuated migration in response to S1P, which is a major chemoattractant present in PB that is responsible for chemoattraction of these cells from the BM microenvironment into circulating blood (12). We propose that pro-mobilizing agents stimulate BM innate immunity cells to release eATP, which occurs in a pannexin-1-channel-dependent manner (12). eATP subsequently activates two crucial receptors from the P2X family (P2X7 and P2X4), which are highly expressed on the surface of HSPCs. eATP acts by engaging these two receptors to activate Nlrp3 inflammasomes. In fact, mice with a defect in the release of eATP into the extracellular space, due to blockade of the pannexin 1 channel with a specific blocking peptide, are poor mobilizers, as are P2X7-KO and P2X4-KO mice (81, 82). These interactions revealed an important pathway involving the release of eATP *via* the pannexin 1 channel, activation of the P2X7 and P2X4 receptors, and Nlrp3 inflammasome activation to stimulate migration of HSPCs. Activation of Nlrp3 inflammasomes in HSPCs and innate immunity cells also leads to the release of other mediators that activate the ComC. We previously reported that ComC cleavage fragments ensure optimal egress of HSPCs into PB (74, 75, 83). The molecular event that occurs after activation of the Nlrp3 inflammasome in HSPCs and promotes and regulates their migration will be discussed later in this review.

### The Involvement of NOD-Like Receptor Family Pyrin Domain-Containing Protein 3 Inflammasomes in Homing and Engraftment of Hematopoietic Stem/Progenitor Cells After Transplantation

Hematopoietic transplantation is preceded by myeloablative conditioning of BM, a pre-transplantation procedure to empty hematopoietic niches and provide space for newly transplanted cells. Myeloablation is performed by employing chemo- or radiotherapy, and the HSPCs that are subsequently infused into the circulation during hematopoietic transplantation sense a homing gradient of BM chemoattractants that promote their navigation to BM hematopoietic niches (61). Here again, as we recently reported, Nlrp3 inflammasome expressed in migrating HSPCs and in cellular components activated in the BM microenvironment plays an important role. Specifically, perturbation of Nlrp3 inflammasome expression directly in HSPCs by the small-molecule inhibitor MCC950 impaired homing and engraftment of these cells after transplantation into control (normal) mice (30). This result has been subsequently reproduced with BM cells from Nlrp3-KO animals (30). This decrease in homing of transplanted HSPCs corresponded to decreased migration of these cells in *in vitro* assays in response to a gradient of the major chemoattractant expressed in the BM microenvironment, SDF-1. In parallel, we also found that Nlrp3 inflammasome expressed in non-hematopoietic cells in the BM microenvironment is also involved in promoting the homing of HSPCs (30). Specifically, as part of the sterile inflammation response in mice due to myeloablative conditioning for transplantation by lethal irradiation, the expression of Nlrp3 inflammasome is upregulated in BM stromal cells (30). In addition, Nlrp3 inflammasome is involved in the upregulation of SDF-1 in response to lethal irradiation. Activated Nlrp3 inflammasome through DAMPs secreted from cells also promotes activation of the ComC, which facilitates engraftment. Accordingly, as mentioned earlier, we have reported that mice that are deficient in the release of ComC cleavage fragments engraft poorly with HSPCs (84–86).

Therefore, as discussed above, Nlrp3 inflammasome is associated with the innate immune response and the state of BM sterile inflammation to promote optimal pharmacological mobilization and enhance homing and engraftment of HSPCs after myeloablative treatment (87, 88).

## The Molecular Events Involved in Nod-Like Receptor Family Pyrin Domain-Containing Protein 3 Inflammasome Activation in Migrating Hematopoietic Stem/Progenitor Cells

**Figure 2:**
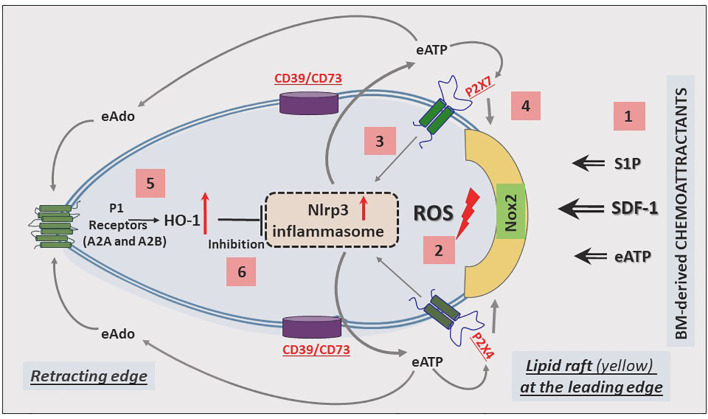
Cell migration-promoting mechanisms at the leading surface/edge and the negative-feedback mechanism at the retracting surface/edge of migrating HSPCs. We propose that in response to BM chemoattractants, HSPCs activate Nox2, which is a membrane lipid raft-associated enzyme and a source of ROS [1]. ROS activates the Nlrp3 inflammasome [2], which releases ATP into the extracellular space surrounding HSPCs [3]. In a positive-feedback mechanism, extracellular ATP (eATP) activates the Nlrp3 inflammasome and membrane lipid raft formation so that cells more robustly respond to BM chemoattractants [4]. In a negative-feedback mechanism, eATP is converted by the cell surface-expressed ectonucleotodases (CD39 and CD73) into extracellular adenosine (eAdo), which *via* the P1 receptors (A2a, A2b) activates heme oxygenase 1 (HO-1) [5], a negative regulator of the Nlrp3 inflammasome [6].

The migration of cells in response to gradients of chemoattractants is based on cell polarization, which results in the accumulation of a specific set of receptors and intracellular signaling molecules at the leading and receding surfaces of the cell. Mathematical modeling suggests that these coordinated chemotactic processes require the assembly of cell migration-stimulating receptors and signaling molecules at the front surface and cell migration-inhibiting receptors and factors at the back surface of the migrating cell (89). This model has been well established for the involvement of autocrine/paracrine-secreted eATP in migrating neutrophils in response to a C5a gradient (90), and we have invoked it in the context of HSPC migration (**Figure 2**).

Based on our published results, the Nlrp3 inflammasome has emerged as a central orchestrator of HSPC trafficking and the purinergic signaling pathway, involving eATP signaling *via* the P2X7 and P2X4 receptors, and seems to be an initiating event during the mobilization of HSPCs. In this process, eATP is released initially from activated and stressed innate immunity cells exposed to pro-mobilizing agents and released in an autocrine-dependent manner from the activated HSPCs themselves. It is also released from cells in the BM microenvironment damaged by myeloablative conditioning for transplantation. **Figure 2** shows the molecular events that lead to the migration of HSPCs: from initial activation to amplification, and to maintaining intracellular Nlrp3 activity, as well as decreasing its activation.

The most important BM-homing chemoattractant is SDF-1 (91–93); however, it is also supported by stromal-derived S1P and eATP, which are released in response to myeloablative treatment (94, 95). These factors, as depicted in **Figure 2**, force (via a cell membrane-expressed nicotinamide adenine dinucleotide phosphate oxidase [NADPH oxidase] isoform known as NOX2) the release of reactive oxygen species (ROS), which activate Nlrp3 inflammasome (5, 96, 97). In fact, there are two sources of ROS: *i)* cell membrane-derived and *ii)* mitochondria-derived ROS, which both strongly activate Nlrp3 inflammasome. As a result of this activation, cells may release several DAMPs, including eATP, that potentiate its activation in an autocrine-dependent manner. Another DAMP, high mobility group box 1 (HMGB1), may activate the classical pathway of the ComC after binding to C1q (98). In fact, we observed this phenomenon in mice injected with HMGB1 protein (12). Hyperactivation of Nlrp3 inflammasomes in innate immunity cells may lead to pyroptosis, the release of proteolytic enzymes from these cells, changes in membrane lipid composition, and leakage of ROS. All this together changes the hematopoietic microenvironment and, together with the release of HMGB1 protein, potentiates activation of the ComC. As mentioned above, ComC cleavage fragments, such as the anaphylatoxins C3a and C5a as well as the non-lytic C5b-C9 complex (MAC), may, in turn, activate the Nlrp3 inflammasome. In addition to DAMPs, active forms of interleukin-1β and interleukin-18 are also released from innate immunity cells and HSPCs, which again may employ an autocrine positive feedback loop to activate the Nlrp3 inflammasome (99). Nlrp3 inflammasome may also be triggered by mitochondrial DNA (mtDNA). It has been nicely demonstrated, for example, that Nlrp3 inflammasome may be activated by monosodium urate-induced mtDNA in Toll-like receptor 9 (TLR9) - dependent manner (100). mtDNA may also drive Nlrp3 inflammasome-dependent aging (101).

Nevertheless, the most important autocrine effector stimulating Nlrp3 inflammasome-dependent migration of HSPCs is eATP, which promotes the formation of membrane lipid rafts that include the CXCR4 receptor for SDF-1 at the leading surface of migrating cells (**Figure 2**). When embedded in membrane lipid rafts in this manner, the CXCR4 receptor responds much more strongly to an SDF-1 gradient (102). A defect in the release of eATP from cells, as seen after pannexin 1 channel blockade, leads to impaired membrane lipid raft formation and reduced migration of HSPCs in response to an SDF-1 gradient (30). A similar effect is achieved by depletion of eATP by pretreatment with apyrase, an enzyme that degrades in extracellular space autocrine-secreted eATP (82).

**Figure 3:**
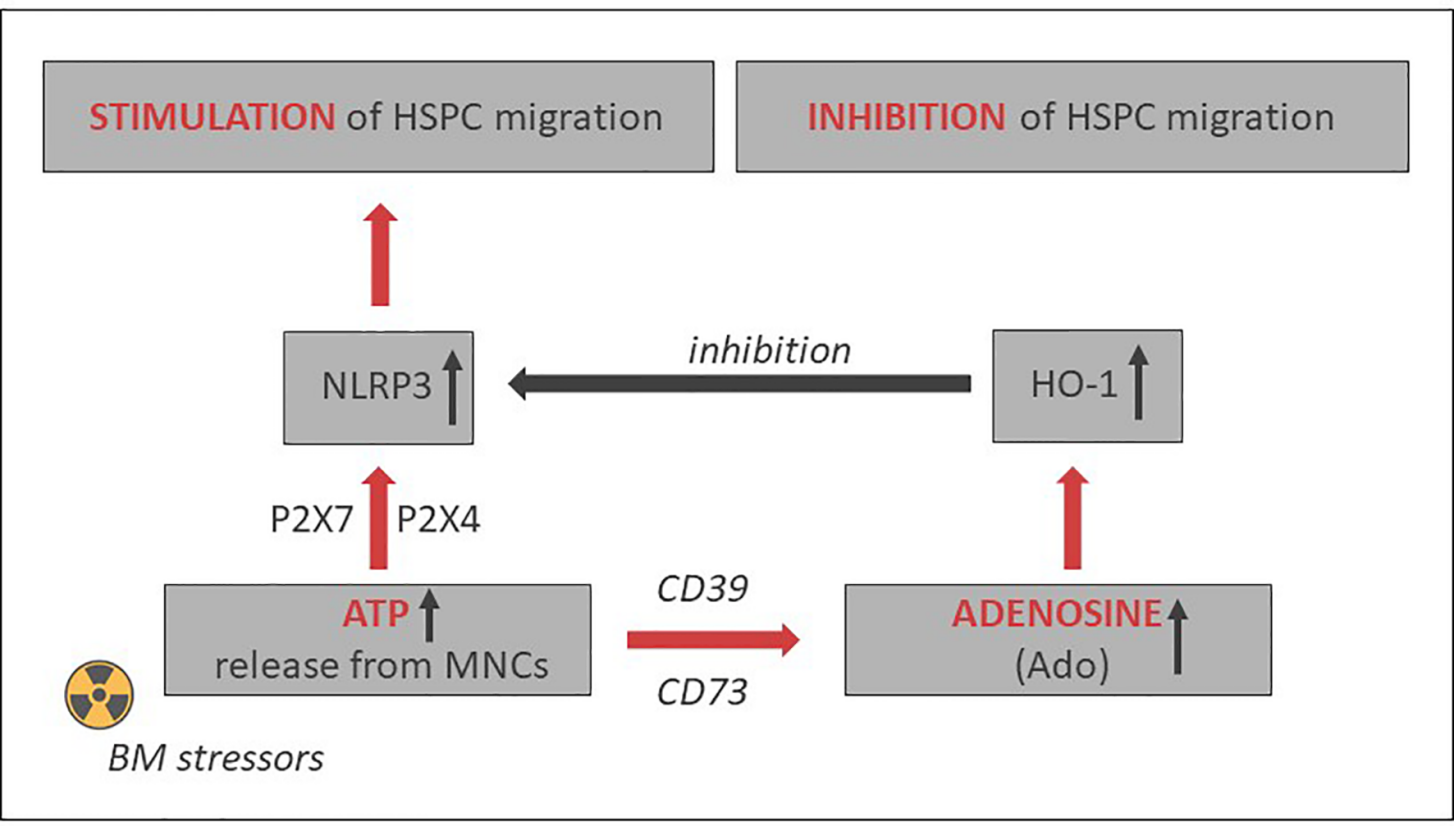
Opposing positive-negative (“Yin–Yang”) effects of eATP and eAdo on stem cell trafficking. While eATP promotes cell migration by activating the Nlrp3 inflammasome, eAdo-induced intracellular heme oxygenase 1 (HO-1) inhibits the Nlrp3 inflammasome and stem cell trafficking. A similar negative effect may have inducible nitric oxide synthetase (iNOS) (103).

As depicted in **Figures 2** and **3**, this Nlrp3-mediated mechanism regulating migration of HSPCs is attenuated by the eATP metabolite extracellular adenosine (eAdo). To explain this finding, eATP is processed by two HSPC surface-expressed ectonucleotidases, CD39 and CD73, to eAdo, which, by engaging specific eAdo signaling receptors at the retracting surface, inhibits the migration of cells (82). To support this eAdo has been already reported to inhibit migration of granulocytes and lymphocytes (104, 105). Our results indicate that HSPCs highly express two out of four members of the family of eAdo receptors, namely A2a and A2b. We are currently investigating which of these two receptors is involved in the inhibitory effects on eAdo migration. We already know that this effect is mediated by the upregulation of intracellular heme oxygenase 1 (HO-1) (106). This anti-inflammatory enzyme is a well-known inhibitor of the Nlrp3 inflammasome and, as we reported, an inhibitor of cell migration (103). Specifically, we reported that HO-1 activators decrease migration of HSPCs, inhibit membrane lipid raft formation at the leading surface of migrating cells, and negatively affect the homing and engraftment of HSPCs (107). These results were subsequently reproduced using HSPCs from HO-1-KO animals.

## Potential Strategies to Positively Modulate Nod-Like Receptor Family Pyrin Domain-Containing Protein 3 Inflammasome Expression For Hematopoietic Transplantations

Based on the aforementioned, the Nlrp3 inflammasome may become an important target to enhance mobilization, homing, and engraftment of HSPCs. **Figure 3** delineates the involvement of the Nlrp3 inflammasome in HSPC trafficking in response to the SDF-1 gradient and shows the effects of positive (eATP) and negative (eAdo) purinergic signaling mediators. There are several potential strategies to increase the normal expression of Nlrp3 inflammasome in HSPCs and in the BM microenvironment that will be briefly presented below.

### To Optimize Potential Strategies to Positively Modulate Nod-Like Receptor Family Pyrin Domain-Containing Protein 3 Inflammasome Expression for Hematopoietic Transplantations Inflammasome Activity During Mobilization of Hematopoietic Stem/Progenitor Cells

There is a need to optimize currently available HSPC mobilization strategies, as a significant percentage of patients are deemed poor mobilizers (62, 108, 109). As mentioned above, mice that are depleted of Gram-negative intestinal bacteria are poor mobilizers (78). This can be explained by the fact that LPS absorbed from the gastrointestinal tract is crucial for the priming of Nlrp3 inflammasomes at the cellular level. This effect is mediated at the transcriptional level by Toll-like receptor 4 (TLR4) in an NFκ-B-dependent manner and by TLR4-mediated phosphorylation of Nlrp3 components (68, 110, 111). Activation of Nlrp3 inflammasomes can be achieved by *in vivo* administration of potent nontoxic activators of the P2X7 and P2X4 receptors, such as 2′(3′)-O-(4-benzoylbenzoyl) adenosine 5′-triphosphate (BzATP), a more potent agonist of the P2X7 receptor than eATP; and ivermectin, a potent agonist of the P2X4 receptor (112, 113). Another strategy is to take advantage of nigericin, which is an antibiotic derived from *Streptomyces hygroscopicus* and is a known potent activator of the Nlrp3 inflammasome by inducing K^+^ efflux, which is a crucial signal for activation of this protein complex (114). Our preliminary results indicate that nigericin at nontoxic doses promotes mobilization of HSPCs (12). Moreover, taking into account the adverse effects of eAdo on the egress of HSPCs from their niches, we can employ the eAdo receptor-blocking agents MSX-2/MSX-3 or PSB-603 or inhibitors of eAdo-induced endogenous heme oxygenase 1 (HO-1). These experimental strategies are ready to be tested in large-animal models.

### To Optimize Nod-Like Receptor Family Pyrin Domain-Containing Protein 3 Inflammasome Expression for Hematopoietic Transplantations Inflammasome Expression in Transplanted Hematopoietic Stem/Progenitor Cells

**Figure 4:**
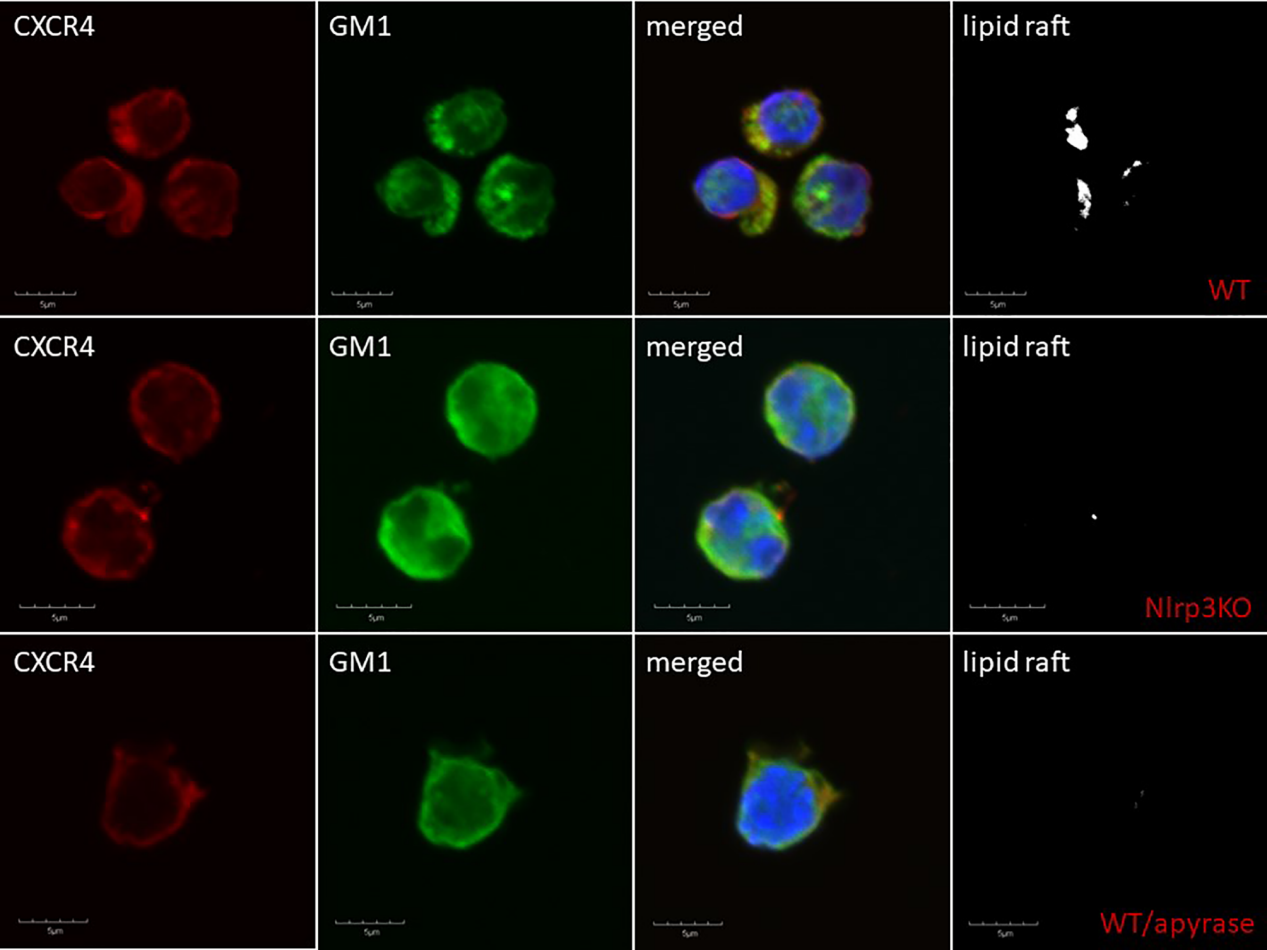
Confocal analysis of membrane lipid rafts in purified murine SKL cells. Representative images of SKL cells sorted from WT BM, stimulated with SDF-1 (50 ng/ml) and LL-37 (2.5 μg/ml); stained with cholera toxin subunit B (a lipid raft marker) conjugated with FITC and rat anti-mouse CXCR4, followed by anti-rat Alexa Fluor 594; and evaluated by confocal microscopy for the formation of membrane lipid rafts. Lipid rafts were formed in SKL cells (upper panel) but not in SKL cells isolated from Nlrp3-KO (middle panel) or cells exposed to apyrase (50 U/ml) lower panel. Representative pictures are shown.

Delayed engraftment of HSPCs, or even failure to engraft, is still a significant problem, mainly if the number of HSPCs is limited (e.g., availability of only a single umbilical cord blood unit, poor mobilizers, poor BM harvest) (109, 115). Therefore, strategies to enhance the homing of transplanted HSPCs by manipulating *ex vivo* their seeding efficiency potential in BM is a significant clinical problem. This can be solved by enhancing lipid raft formation in HSPCs after *ex vivo* exposure of these cells to eATP (30). As depicted in **Figure 2**, the migration of cells in a gradient of chemoattractant is based on cell polarization, which results in the accumulation of a specific set of migration-promoting receptors and intracellular signaling molecules associated with membrane lipid rafts at the leading surface and receptors that negatively affect this process at the receding surface of the cell. Our preliminary results indicate that the Nlrp3 inflammasome plays an important role in increasing chemotactic responsiveness in the migration of HSPCs in an SDF-1 gradient because, as reported previously, HSPCs from Nlrp3-KO mice show *i)* defective migration in response to SDF-1, S1P, and eATP gradients and i*i)* engraft more poorly than syngeneic WT cells after transplantation (30). This impaired migration, homing, and engraftment depends on decreased autocrine secretion of eATP on the leading surface and defective formation of membrane lipid rafts. Based on this finding, we may try to increase the responsiveness of HSPCs to homing gradients by stimulating the Nlrp3 inflammasome by *ex vivo* activation of the P2X4 and P2X7 receptors on the surface of HSPCs by employing (as mentioned in a previous section) BzATP, a potent nontoxic agonist of these receptors, and ivermectin, a potent agonist of P2X4. The Nlrp3 inflammasome can also be activated directly with nigericin (80). Another strategy would be to inhibit eATP conversion to migration-inhibiting eAdo by blocking CD39 and CD73 on the surface of HSPCs *ex vivo* before transplantation. eATP is metabolized to eAdo, which, as we demonstrated, has a negative effect on the migration of HSPCs (82). Therefore, HSPCs could be exposed *ex vivo* to the small-molecule inhibitors ARL67156 and AMPCP of CD39 and CD73, respectively. We could also employ, as mentioned above, inhibitors of the eAdo receptors. Moreover, our recent research indicates that the Nlrp3 inflammasome could also be activated in HSPCs by exposure to prostaglandin E2 (PGE2). In fact, PGE2 is currently employed in the clinic to facilitate homing and engraftment of HSPCs (116, 117). **Figure 4** delineates that SDF-1/LL-37 induced lipid raft formation containing CXCR4 homing receptor occurs in wild type murine HSPCs cells, but it does not occur in HSPCs cells isolated from Nlrp3-inflammasome-KO mice or cells exposed to apyrase (30). This latter result with apyrase confirms an autocrine role of eATP released in Nlrp3-inflammasome-dependent manner in promoting lipid raft formation, as shown in **Figure 2**.

### To Optimize Activation of the Nod-Like Receptor Family Pyrin Domain-Containing Protein 3 Inflammasome Expression for Hematopoietic Transplantations Inflammasome in Myeloablated Bone Marrow of the Transplantation recipient

Delayed engraftment of HSPCs, or even failure to engraft, could also be a result of defective homing properties of the BM microenvironment. There are several strategies possible to enhance homing and engraftment of transplanted HSPCs by modulating the BM microenvironment in transplantation recipients by improving the biological effectiveness of the eATP–Nlrp3 inflammasome axis and inhibiting the negative effects of eAdo. Again, one can consider employing nontoxic doses of small-molecule inhibitors of the CD39 and CD73 enzymes (ARL67156 and AMPCP, respectively), as these enzymes are involved in the generation of eAdo in the extracellular space, and inhibitors of the A2a and A2b P1 receptors for eAdo (ANR94 and PSB 603, respectively). In recently published work, it has been demonstrated that proper expression of Nlrp3 inflammasome in BM microenvironment is also involved in facilitating mobilization and homing as well as engraftment of HSPCs (30). This has been confirmed in normal chimeric mice transplanted with BMMNC from Nlrp3-KO and Nlrp3-KO animals bearing control BMMMNC. We are currently trying to address in more detail the role of the Nlrp3-KO microenvironment in these defects.

## The Potential Role of the Nod-Like Receptor Family Pyrin Domain-Containing Protein 3 Inflammasome Expression for Hematopoietic Transplantations Inflammasome In *Ex Vivo* and *In Vivo* Expansion of Hematopoietic Stem/Progenitor Cells

Expansion of HSPCs *ex vivo* is important for enriching hematopoietic grafts for these cells or, after transplantation *in vivo*, speeding repopulation of myeloablated BM with transplanted cells. It has been proposed that Nlrp3 inflammasome-mediated glucose influx into HSPCs in the developing vertebrate embryo expands murine early-development CD41^+^ HSPCs *in vivo*, and this effect depends on Nlrp3 inflammasome activation and IL-1β release and is suppressed in IL-1β-KO cells (29). Moreover, as stated, the loss of Nlrp3 inflammasome components prevents the proliferation of embryonic HSPCs (29). Interestingly, positive expansion results were obtained with human iPSC-derived hemogenic cells in the presence of Nlrp3 inflammasome activators. This expansion resulted in a significant increase in multilineage hematopoietic colony formation and strongly suggests that the Nlrp3 inflammasome may indeed regulate the expansion of early-development HSPCs (13, 29). These provocative findings, however, require further study to assess whether, in addition to embryonic HSPCs and iPSC-derived hemogenic cells, the Nlrp3 inflammasome may also expand postnatal normal murine and human HSPCs. Based on the aforementioned, stimulation of Nlrp3 inflammasome activity may become an adjuvant strategy to improve *ex vivo* expansion of HSPCs. In support of this possibility, our recent results indicate that Nlrp3-KO mice have ~20% fewer Sca-1^+^Kit^+^Lin^–^ (SKL) HSPCs in the BM than do WT animals (28). Another question is how expansion could be affected by the eATP metabolite eAdo. Somewhat surprisingly, in a zebrafish embryo model, eAdo has been proposed as a positive regulator of hematopoiesis (118). This potential discrepancy between zebrafish and human could be explained by developmental age and species differences, as, in our hands, eAdo affects proliferation of neither murine nor human HSPCs.

The role of Nlrp3 inflammasomes on proliferation and development of HSPCs needs to be better addressed in models of stress-induced hematopoiesis. The question is whether Nlrp3 inflammasome-KO animals will display a defect in hematopoietic recovery from sublethal irradiation. Moreover, if any defects are seen, would they depend on Nlrp3 inflammasome expression in HSPCs or in the BM hematopoietic microenvironment? To address these questions, we are currently performing the appropriate experiments.

## Conclusions

The Nlrp3 inflammasome has become a “rising star” in the study of normal and pathological hematopoiesis, as it has pleiotropic effects in hematopoietic cells. The physiological activation of this intracellular protein complex is crucial to maintaining normal hematopoiesis and normal trafficking of HSPCs, and its hyperactivation leads to cell death by pyroptosis. In this review, we summarized the emerging role of the Nlrp3 inflammasome in connecting purinergic signaling and the responses of innate immunity to direct mobilization, homing, and engraftment of transplanted HSPCs. Potential approaches have also been proposed that are based on the modulation of Nlrp3 inflammasome activity, and that could positively affect outcomes of hematopoietic transplantations. What was not discussed in this review is the fact that prolonged hyperactivation of the Nlrp3 inflammasome is involved in sterile inflammation of BM, which may contribute to certain hematological pathologies, including myelodysplastic syndrome, myeloproliferative neoplasms, leukemia, the onset of graft-versus-host disease (GvHD) after transplantation, and the induction of cytokine storms, as seen as a complication in CAR-T cell therapy or COVID19 infection (reviewed in reference 13). Therefore, more research is needed to define the boundaries of the safety window and to study the transition of Nlrp3 inflammasomes from being beneficial to a detrimental modulator of the hematopoietic system.

## Author Contributions

MR wrote the paper. MK prepared the figures and approved the paper. All authors contributed to the article and approved the submitted version.

## Funding

This work was supported by NIH grants 2R01 DK074720, the Stella and Henry Endowment, and the OPUS grant UMO-2018/29/B/NZ4/01470 to MR.

## Conflict of Interest

The authors declare that the research was conducted in the absence of any commercial or financial relationships that could be construed as a potential conflict of interest.
